# Access and satisfaction with healthcare services among chronic disease patients during the Sudan armed conflict: a cross-sectional study

**DOI:** 10.1186/s13031-025-00703-y

**Published:** 2025-08-11

**Authors:** Sohaib Mohammed Mokhtar Ahmed, Moram Elfadel Abdelrhaman Gasmalha, Ahmed Balla M. Ahmed, Salma Alrawa, Khalid Abusofyan Eljezoli Mohammed, Muhannad Bushra Masaad Ahmed

**Affiliations:** 1 Faculty of Medicine and Health Sciences, University of Gadarif, Gadarif, Sudan; 2 Faulty of Medicine and Health Sciences, Omdurman Islamic University, Omdurman, Sudan; 3 Faculty of Medicine, University of Khartoum, Al-Qasr Street, PO Box: 102, Khartoum, 11111 Sudan; 4 Faculty of Dentistry, University of Gezira, Wad Madani, Sudan

**Keywords:** Healthcare services, Access, Satisfaction, Chronic diseases, Armed conflict, Sudan

## Abstract

**Background:**

The ongoing conflict in Sudan has severely disrupted the healthcare system, leaving millions without access to essential services. Chronic disease patients are among the most affected due to their heightened reliance on consistent and specialized healthcare. This study aimed to assess the level of access to healthcare services and the satisfaction of chronic disease patients during the current war in Sudan.

**Methods:**

A cross-sectional study was conducted among chronic disease patients residing in the safest states of Sudan during the ongoing conflict. Access to healthcare services and patient satisfaction were measured using a structured questionnaire that was specially designed and piloted for the purpose of this study. Descriptive statistics, including median, interquartile range, and percentages, were used to summarize the data. Inferential statistical methods, such as chi-square tests, were then applied to examine associations between socio-demographic factors and healthcare access. Statistical significance was defined as *p* < 0.05 for all analyses.

**Results:**

Among 1116 chronic disease patients, 13.3% never visited a health facility for regular check-ups during the current war, 20.1% reported facilities were over 5 km away by walking distance, 15.9% rarely or never accessed healthcare services during the war, and 22.0% reported a very large effect of the war on their healthcare access. Additionally, 22.3% noted excellent availability of healthcare personnel during the current war, while 18.1% were dissatisfied with the quality of care. Significant associations (*p* < 0.01) were found between the frequency of health facility visits and factors such as income level, ease of access, availability of personnel, satisfaction with care, confidence in providers, and the war’s impact on access.

**Conclusion:**

Chronic disease patients experienced suboptimal follow-up for their conditions, limited access to healthcare services, and a significant proportion expressed dissatisfaction with the quality of care during the current Sudan war. To mitigate these challenges, coordinated efforts are urgently needed to strengthen Sudan’s healthcare system, improve geographic access, and support healthcare providers.

**Supplementary Information:**

The online version contains supplementary material available at 10.1186/s13031-025-00703-y.

## Background

Wars inflict profound and far-reaching damage on public health [1], disrupting healthcare systems, displacing populations, and worsening disease burdens, especially in settings with limited medical resources and poor living conditions [2–4]. Vulnerable groups—such as children, the elderly, and individuals with chronic illnesses—bear a disproportionate share of the burden, as their pre-existing medical needs make them especially susceptible to the healthcare challenges posed by armed conflict [5, 6].

Before the current war in Sudan, non-communicable diseases (NCDs) were already a major public health concern, accounting for 53.9% of all deaths. The most prevalent NCDs included cardiovascular diseases, cancers, respiratory illnesses, and diabetes mellitus [7]. Despite this high disease burden, access to healthcare was limited, with only 70% of the population living within 30 min of a health facility, and 80% within one hour [8].

Since the outbreak of war in April 2023, Sudan’s already fragile healthcare system has been pushed to the brink of collapse, with 70% of facilities in conflict-affected areas no longer operational [9]. These disruptions have severely limited access to essential services, particularly for patients with chronic conditions who require continuous care [10, 11]. The ongoing conflict has escalated into one of the world’s worst humanitarian crises, displacing over 6.8 million people internally and forcing 1.5 million to flee as refugees. Nearly half of the population—approximately 24.8 million people—now relies on humanitarian aid [12].

Evidence from similar crises highlights these challenges. In Ethiopia’s Tigray War, healthcare utilization for chronic diseases dropped dramatically. For instance, Gebrehiwet et al. reported a 94% reduction in follow-up care for type 1 diabetes [13], and another study revealed that 64.6% of chronic disease patients faced treatment interruptions due to service unavailability, medication shortages, transportation challenges, and displacement of healthcare workers. Such disruptions increase morbidity and mortality while imposing significant economic and psychological burdens on affected individuals [14].

Despite the gravity of these challenges, research specifically examining the experiences of chronic disease patients during the ongoing armed conflict in Sudan has been notably scarce. This study aimed to investigate chronic disease patients’ access to and satisfaction with healthcare services amid the conflict.

## Methods

### Study design

This cross-sectional study assessed the level of access to healthcare services and the satisfaction of chronic disease patients during the current war in Sudan. The research followed the Strengthening the Reporting of Observational Studies in Epidemiology (STROBE) guidelines to ensure methodological rigor and transparent reporting of findings.

### Study setting and duration

The study was conducted in some of the safest states in Sudan, including the Red Sea, Kassala, and Gadarif states in eastern Sudan; Northern and River Nile states in northern Sudan; and White Nile state in central Sudan. During the latter half of 2024, when data collection took place, active fighting was concentrated in Khartoum—the capital—along with Gezira, Sennar, parts of White Nile State in central Sudan, most of western Sudan, and large areas of Kordofan. This made the northern and eastern states, along with parts of central Sudan, the relatively safest regions during the study period [15, 16].

This study was conducted across both community and hospital settings, specifically targeting households and healthcare facilities. This approach was chosen to capture a broader perspective on healthcare access and satisfaction, as some patients may not regularly attend facilities. Including both settings allowed us to assess experiences of those actively seeking care and those facing barriers. In hospital settings, participants were recruited from specialized centers and hospital departments managing chronic conditions, including diabetes centers, renal centers and hospitals, oncology centers and hospitals, as well as inpatient wards for diseases like tuberculosis and asthma, and general outpatient and inpatient departments. In community settings, data collectors identified individuals with chronic illnesses through personal networks—approaching relatives, neighbors, and acquaintances known to have chronic conditions. Participants were also recruited from temporary accommodations/shelters and internally displaced/refugee camps, and community referrals were used to expand reach, especially in the absence of official patient records due to the ongoing conflict. Data collection took place from September 16 to December 2, 2024.

### Participants and sampling

The study targeted individuals aged 18 years or older diagnosed with a chronic disease, such as diabetes, hypertension, cardiovascular diseases, respiratory conditions, or other long-term illnesses [17]. Only individuals who had been living with these conditions for at least one year were included, to ensure they had sufficient experience with their illness to accurately assess their access to healthcare and level of satisfaction. Other inclusion criteria required participants to provide written informed consent and have the cognitive ability to complete the interview.

The sample size was determined using the Cochrane formula, given the absence of official population records in Sudan. Assuming a population proportion of 50%, a 5% margin of error, and a 95% confidence interval, the minimum required sample size was calculated at 385 participants. We aimed to include as many patients with chronic diseases as possible during the data collection period, ultimately recruiting 1116 individuals. This large sample size helps enhance the diversity of the sample and allows for more precise estimation of outcomes within the study context.

Due to the challenges posed by the ongoing conflict in Sudan, which restricted access to official patient records, convenience sampling was utilized.

### Data collection procedures

Data collection was carried out by trained medical students who administered a structured, face-to-face interviewer-administered questionnaire to participants using electronic devices via the KoboCollect application. The data were collected electronically in real time—not on paper—to ensure accuracy and efficiency. The interviews were conducted in a confidential manner to encourage honest and accurate responses while maintaining participants’ privacy. All data collectors received training to ensure consistency, accuracy, and adherence to ethical standards during the data collection process.

### Data collection tools

The data collection tool used in this study is structured into two sections. The first section gathers basic demographic details such as age, sex, marital status, highest educational level, employment status, living situation, internal displacement status, income level (self-reported), and chronic disease diagnoses, with a total of 9 questions. The second section includes 7 questions that assess the frequency and ease of healthcare access, healthcare staff availability and patient confidence in their care, satisfaction with care, and the impact of the ongoing conflict on healthcare access (Supplementary file). The questionnaire was designed by the authors specifically for this study and was administered in Arabic to participants, as Arabic is the most widely spoken language in Sudan. After data collection, the questionnaire was translated into English by the authors for reporting and publication purposes. The English version was reviewed by an expert to ensure accuracy and consistency. The questionnaire was piloted among 30 participants with chronic diseases recruited from across the selected states, who were later excluded from the final sample. Their inquiries and feedback were addressed in the final version of the questionnaire, resulting in minor corrections to wording and phrasing, with no major structural changes.

### Data analysis

The dataset was first extracted from the KoboCollect platform into Excel before being imported into the Statistical Package for the Social Sciences (SPSS) software version 27 for analysis. Descriptive statistics, such as median, interquartile range, and percentages, were used to summarize socio-demographic characteristics and healthcare utilization patterns. Inferential statistical methods, including chi-square tests, were employed to examine associations of socio-demographic factors with healthcare access. Statistical significance was set at *p* < 0.05 for all analyses.

## Results

### Socio-demographic characteristics of participants

A total of 1116 participants were enrolled based on the predefined inclusion criteria. Recruitment was conducted by directly assessing eligibility during participant encounters; therefore, the number of individuals approached but not meeting inclusion criteria was not recorded. The median age of the participants was 44 years (IQR = 29). The majority of participants were female (60.1%). Education levels varied, with 48.8% holding a bachelor’s degree and 22.4% having completed high school. Employment status was diverse, with 38.7% of participants unemployed. Regarding housing, 48.6% lived with family or friends, 22.6% resided in rented houses, and 2.3% lived in IDP/refugee camps. Internally displaced individuals made up 58.3% of the participants. Income levels were reported as low by 13.9% and high by 2.1% (Table 1).

**Table 1:** Socio-demographic characteristics of participants (*N* = 1116)

Socio-demographic Factors	Overall (*N* = 1116)
**Age**	
Median (IQR)	44 (29)
**Sex**	
Female	671 (60.1%)
Male	445 (39.9%)
**Marital status**	
Married	608 (54.5%)
Single	384 (34.4%)
Divorced	38 (3.4%)
Widow	86 (7.7%)
**Highest educational level **	
Illiterate	49 (4.4%)
Primary school	140 (12.5%)
High school	250 (22.4%)
bachelor’s degree	545 (48.8%)
Higher studies	72 (6.5%)
Informal education (Khalwa)	60 (5.4%)
**Employment status**	
Student	256 (23.0%)
Unemployed	432 (38.7%)
Self-employed	164 (14.7%)
Government employee	171 (15.3%)
Private sector employee	92 (8.3%)
**Living situation**	
With family and friends	542 (48.6%)
Own house	203 (18.2%)
Rented house	252 (22.6%)
Temporary Accommodation / Shelter	93 (8.3%)
IDP/Refugee Camp	26 (2.3%)
**Internal displacement**	
No	465 (41.7%)
Yes	650 (58.3%)
**Income level**	
Low	155 (13.9%)
Below average	201 (18.0%)
Average	611 (54.7%)
More than average	126 (11.3%)
High	23 (2.1%)

### Chronic diseases

**Fig. 1:**
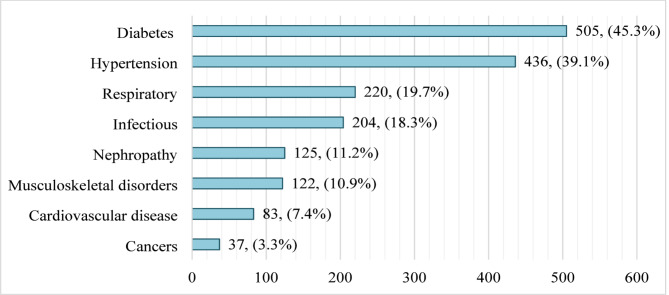
Prevalence of chronic diseases among participants. Note: Participants were allowed to report more than one comorbidity

The most common chronic condition among participants was diabetes mellitus, affecting 45.3% of the study population, followed by systemic hypertension at 39.1%. Respiratory diseases were reported in 19.7% of participants, while infectious diseases, including tuberculosis and hepatitis, accounted for 18.3%. The least reported condition was cancer, affecting 3.3% of participants (Fig. 1).

### Access and satisfaction with healthcare services

Among the participants, 13.3% reported never visiting a health facility for regular check-ups during the current war, while 20.6% visited rarely (once a year or less). Access to healthcare facilities during the current war was very easy for 15.7%, while 38.7% had moderate access (1–5 km from their residence). However, 8.5% reported very hard access (more than 10 km). Regarding healthcare access during the war when needed, 3.9% reported never having access, 12.0% rarely, and 19.1% always. In terms of healthcare personnel availability during the current war, 3.6% rated it as very weak, while 30.8% and 22.3% rated availability as good and excellent, respectively (Table 2).

Satisfaction with healthcare services during the war showed that 3.0% were very unsatisfied, while 17.6% were very satisfied. The ongoing conflict had a significant impact on healthcare access, with 10.6% reporting no effect, and 30.7% and 22.0% reporting large and very large effects, respectively (Table 3).

**Table 2:** Access to healthcare services among participants

Question	Response	*N* (%)
**How often do you visit a health facility for regular check-ups?**	Never	148 (13.3%)
	Rarely (once a year or less)	230 (20.6%)
	Sometimes (every 4–6 months)	240 (21.5%)
	Frequently (every 2–3 months)	293 (26.3%)
	Very frequently (monthly or more)	205 (18.4%)
**How easy is it to get to the nearest health facility from your place of residence?**	Very easy (within walking distance)	175 (15.7%)
	Easy (less than 1 km)	284 (25.5%)
	Moderate (1–5 km)	432 (38.7%)
	Hard (5–10 km)	129 (11.6%)
	Very hard (more than 10 km)	95 (8.5%)
**How often do you have access to healthcare services when you need them?**	Never	43 (3.9%)
	Rarely	134 (12.0%)
	Sometimes	383 (34.3%)
	Often	343 (30.7%)
	Always	213 (19.1%)
**How do you assess the availability of healthcare personnel (doctors**,** nurses**,** etc.) at the facility you visit?**	Very weak	40 (3.6%)
	Weak	122 (10.9%)
	Moderate	361 (32.3%)
	Good	344 (30.8%)
	Excellent	249 (22.3%)

**Table 3:** Satisfaction with healthcare services among participants

Question	Response	*N* (%)
**How satisfied are you with the quality of care provided during your healthcare visits?**	Very unsatisfied	34 (3.0%)
	Unsatisfied	168 (15.1%)
	Neutral	286 (25.6%)
	Satisfied	432 (38.7%)
	Very satisfied	196 (17.6%)
**How confident are you in your healthcare providers’ ability to manage your health?**	Not confident	37 (3.3%)
	Slightly confident	181 (16.2%)
	Neutral	262 (23.5%)
	Confident	443 (39.7%)
	Very confident	193 (17.3%)
**To what extent has the conflict affected your ability to access healthcare services?**	No effect	118 (10.6%)
	Little effect	186 (16.7%)
	Moderate effect	224 (20.1%)
	Large effect	343 (30.7%)
	Very large effect	245 (22.0%)

During the current Sudan war, the frequency of health facility visits for regular check-ups showed significant associations with income level, ease of access to health facilities, access to healthcare services, availability of healthcare personnel, satisfaction with care quality, confidence in healthcare providers, and the impact of conflict on healthcare access (Table 4). Individuals with higher income levels were more likely to visit frequently, while those with lower incomes reported less frequent visits (χ²=41.47, *p* < 0.01). Proximity to healthcare facilities facilitated more frequent visits, with easier access significantly associated with higher visit frequency (χ²=127.07, *p* < 0.01). Access to healthcare services when needed was also strongly associated, as those with consistent access reported visiting more regularly (χ²=565.62, *p* < 0.01). The availability of healthcare personnel and confidence in their abilities positively influenced visit frequency (χ²=246.68, *p* < 0.01 and χ²=186.26, *p* < 0.01, respectively). Satisfaction with care quality encouraged more frequent visits (χ²=198.24, *p* < 0.01). Additionally, the conflict had a significant impact, with individuals reporting larger disruptions to healthcare access visiting less frequently (χ²=94.45, *p* < 0.01). Surprisingly, internal displacement showed no significant association with the frequency of health facility visits (χ²=0.80, *p* = 0.942), although the proportion of visits among internally displaced individuals increased slightly.

**Table 4:** Association between demographic and conflict-related factors with healthcare access frequency

Variable	Never (*N* = 148)	Rarely (*N* = 230)	Sometimes (*N* = 240)	Frequently (*N* = 293)	Too Much (*N* = 205)	Total (*N* = 1116)	χ², *p*-value
**Internal displacement**							χ²=0.80, *p* = 0.942
No	66 (44.6%)	97 (42.2%)	97 (40.6%)	119 (40.6%)	86 (42.0%)	465 (41.7%)	
Yes	82 (55.4%)	133 (57.8%)	142 (59.4%)	174 (59.4%)	119 (58.0%)	650 (58.3%)	
**Income level**							χ²=41.47, *p* < 0.01
Low	26 (17.6%)	19 (8.3%)	31 (12.9%)	52 (17.7%)	27 (13.2%)	155 (13.9%)	
Below average	25 (16.9%)	60 (26.1%)	35 (14.6%)	45 (15.4%)	36 (17.6%)	201 (18.0%)	
Average	79 (53.4%)	129 (56.1%)	141 (58.8%)	150 (51.2%)	112 (54.6%)	611 (54.7%)	
More than average	15 (10.1%)	17 (7.4%)	32 (13.3%)	42 (14.3%)	20 (9.8%)	126 (11.3%)	
High	3 (2.0%)	5 (2.2%)	1 (0.4%)	4 (1.4%)	10 (4.9%)	23 (2.1%)	
**Ease of access to nearest health facility**							χ²=127.07, *p* < 0.01
Very easy (walking distance)	29 (19.6%)	31 (13.5%)	29 (12.1%)	30 (10.2%)	56 (27.3%)	175 (15.7%)	
Easy (< 1 km)	34 (23.0%)	47 (20.4%)	57 (23.8%)	73 (24.9%)	73 (35.6%)	284 (25.5%)	
Moderate (1–5 km)	50 (33.8%)	80 (34.8%)	107 (44.8%)	155 (52.9%)	40 (19.5%)	432 (38.7%)	
Hard (5–10 km)	18 (12.2%)	48 (20.9%)	28 (11.7%)	27 (9.2%)	8 (3.9%)	129 (11.6%)	
Very hard (> 10 km)	17 (11.5%)	24 (10.4%)	18 (7.5%)	8 (2.7%)	28 (13.7%)	95 (8.5%)	
**Access to healthcare when needed**							χ²=565.62, *p* < 0.01
Never	27 (18.2%)	9 (3.9%)	2 (0.8%)	3 (1.0%)	2 (1.0%)	43 (3.9%)	
Rarely	25 (16.9%)	67 (29.1%)	27 (11.2%)	11 (3.8%)	4 (2.0%)	134 (12.0%)	
Sometimes	53 (35.8%)	97 (42.2%)	145 (60.4%)	65 (22.2%)	23 (11.2%)	383 (34.3%)	
Often	21 (14.2%)	37 (16.1%)	42 (17.5%)	176 (60.1%)	67 (32.7%)	343 (30.7%)	
Always	22 (14.9%)	20 (8.7%)	24 (10.0%)	38 (13.0%)	109 (53.2%)	213 (19.1%)	
**Availability of healthcare personnel**							χ²=246.68, *p* < 0.01
Very weak	24 (16.2%)	9 (3.9%)	4 (1.7%)	1 (0.3%)	2 (1.0%)	40 (3.6%)	
Weak	21 (14.2%)	44 (19.1%)	26 (10.8%)	27 (9.2%)	4 (2.0%)	122 (10.9%)	
Moderate	42 (28.4%)	81 (35.2%)	89 (37.1%)	118 (40.3%)	31 (15.1%)	361 (32.3%)	
Good	39 (26.4%)	70 (30.4%)	84 (35.0%)	90 (30.7%)	61 (29.8%)	344 (30.8%)	
Excellent	22 (14.9%)	26 (11.3%)	37 (15.4%)	57 (19.5%)	107 (52.2%)	249 (22.3%)	
**Satisfaction with quality of care**							χ²=198.24, *p* < 0.01
Very unsatisfied	17 (11.5%)	10 (4.3%)	5 (2.1%)	0 (0.0%)	2 (1.0%)	34 (3.0%)	
Unsatisfied	24 (16.2%)	46 (20.0%)	37 (15.4%)	50 (17.1%)	11 (5.4%)	168 (15.1%)	
Neutral	52 (35.1%)	66 (28.7%)	71 (29.6%)	77 (26.3%)	20 (9.8%)	286 (25.6%)	
Satisfied	41 (27.7%)	93 (40.4%)	92 (38.3%)	122 (41.6%)	84 (41.0%)	432 (38.7%)	
Very satisfied	14 (9.5%)	15 (6.5%)	35 (14.6%)	44 (15.0%)	88 (42.9%)	196 (17.6%)	
**Confidence in providers’ ability**							χ²=186.26, *p* < 0.01
Not confident	16 (10.8%)	15 (6.5%)	5 (2.1%)	1 (0.3%)	0 (0.0%)	37 (3.3%)	
Slightly confident	34 (23.0%)	55 (23.9%)	39 (16.2%)	36 (12.3%)	17 (8.3%)	181 (16.2%)	
Neutral	36 (24.3%)	48 (20.9%)	57 (23.8%)	94 (32.1%)	27 (13.2%)	262 (23.5%)	
Confident	49 (33.1%)	92 (40.0%)	108 (45.0%)	119 (40.6%)	75 (36.6%)	443 (39.7%)	
Very confident	13 (8.8%)	20 (8.7%)	31 (12.9%)	43 (14.7%)	86 (42.0%)	193 (17.3%)	
**Conflict’s effect on access**							χ²=94.45, *p* < 0.01
No effect	30 (20.3%)	23 (10.0%)	27 (11.2%)	11 (3.8%)	27 (13.2%)	118 (10.6%)	
Little effect	21 (14.2%)	54 (23.5%)	50 (20.8%)	36 (12.3%)	25 (12.2%)	186 (16.7%)	
Moderate effect	32 (21.6%)	62 (27.0%)	49 (20.4%)	60 (20.5%)	21 (10.2%)	224 (20.1%)	
Large effect	35 (23.6%)	54 (23.5%)	64 (26.7%)	125 (42.7%)	65 (31.7%)	343 (30.7%)	
Very large effect	30 (20.3%)	37 (16.1%)	50 (20.8%)	61 (20.8%)	67 (32.7%)	245 (22.0%)	

## Discussion

Non-communicable diseases represent a significant burden on populations affected by humanitarian crises and disasters [18]. In our study, over one-third of participants reported visiting health facilities only once a year or not at all. More than half indicated that the ongoing conflict had negatively impacted their ability to access healthcare services, while one-fifth expressed dissatisfaction with the quality of care received.

We found that more than a third of participants with chronic diseases never visit health facilities during the current Sudan war, or they visit only once per year or after more than a year has passed. This suggests suboptimal follow-up [19, 20] and puts the patients at increased risk of complications and higher need for hospitalization [21, 22]. The low number of visits may be due to poor counseling for chronic disease patients in Sudan, as they are not properly referred to primary healthcare centers after discharge [23], beside other difficulties like financial constraints and living far from health facilities, where the main hospitals are concentrated in urban centers, adding to the challenges of transportation. We found no significant association between displacement status and access to healthcare services. This contrasts with existing literature, which often links displacement to reduced access to healthcare services [24]. However, a possible explanation for our finding is the nature of displacement in our study context. Most of the internally displaced individuals were not living in camps but had integrated into host communities within the safer states of Sudan. This community-based integration may have reduced the healthcare access gap between displaced and non-displaced individuals. Additionally, the overall strain on the healthcare system during the conflict may have led to widespread barriers that affected all individuals similarly, regardless of displacement status.

Almost a fifth of participants reported that the centers were more than 5 km away by walking distance, which is considered inaccessible according to the United Nations High Commissioner for Refugees (UNHCR) [25]. However, this level of access is better than the general access in Sudan, where only 53% of individuals live within 5 km of health centers. Nonetheless, it is comparable to urban areas, where 88% of residents live within 5 km of healthcare facilities [26]. The geographic access is significantly associated with utilization of healthcare services consistent with previous literature [27, 28]. The Sudanese Ministry of Health has invested in primary healthcare to improve access and provide essential services, but these efforts still fall short of the planned targets [29].

Participants reported that healthcare practitioners were available at healthcare facilities during the war. However, nearly one-fifth of participants had below-average confidence in healthcare practitioners. Although the study did not explore the specific reasons behind this low confidence, several contextual factors may contribute to it, such as the shortage of specialists, limited equipment, and the high turnover of staff due to migration or security threats. The Sudanese conflict has worsened the ongoing migration crisis of qualified Sudanese doctors seeking work abroad [30], which may affect the perceived quality of care. In such contexts, patients may rely more on informal healthcare sources, home remedies, or delay seeking care altogether—practices that warrant further investigation. A previous study from Sudan found a significant association between the presence of qualified staff at healthcare facilities and the likelihood of seeking medical services [31]. There is a pressing need to improve the retention of qualified healthcare workers to strengthen the healthcare system [32]. Future research should explore patients’ coping strategies when confidence in healthcare providers is low and identify community-based alternatives that emerge in crisis settings. Such findings would be essential for informing policy and decision-making regarding workforce training, deployment, and support.

A fifth of participants were dissatisfied with the quality of care during the war. This percentage of dissatisfaction is higher than what is reported in Lebanon [33], but lower than in Tanzania [34]. The dissatisfaction may be attributed to limited physical access to health facilities, low confidence in healthcare providers’ abilities, and poor availability of healthcare personnel. These issues were likely exacerbated by the ongoing armed conflict, which disrupted healthcare delivery and further strained an already fragile health system. There was a significant association between patients’ satisfaction and the frequency of utilization of healthcare services. Patients’ satisfaction is a key determinant of treatment uptake, adherence, and health-related behavior [35, 36].

More than half of the patients reported a significant negative impact of the conflict on their access to healthcare services. This reflects the broader challenges faced by the population, including damaged health infrastructure, closure of public facilities, medication shortages, and insecurity limiting movement. Importantly, we found a significant association between the perceived impact of the conflict and healthcare access frequency, where individuals reporting greater disruption visited health facilities less often. The Sudanese conflict has disrupted medical services by damaging infrastructure, rendering hospitals and medical centers nonfunctional [37], and causing a severe shortage of medications [38]. There is a need for investment in the Sudanese healthcare system from the international community to mitigate the effects of the war. Additionally, collaboration between local stakeholders is essential for the effective coordination of available resources. Community-led interventions should also be encouraged to raise funds for primary healthcare centers and help alleviate the impact of the conflict [39].

While the association between the factors discussed and healthcare accessibility is well-documented in non-crisis settings, their persistence in the context of ongoing armed conflict reinforces the severity of access challenges faced by affected populations. Rather than presenting new associations, our findings underline how these well-known barriers are sustained—and in some cases worsened—during times of war. Highlighting their continued impact in this context is important for guiding context-specific interventions and strengthening health system resilience in humanitarian crises.

This study provides valuable insights into chronic disease management in safe areas during the Sudanese military conflict. Due to the challenges caused by humanitarian settings, studying chronic disease management in active war zones is difficult; however, one can infer a worse situation.

This study has several limitations. The use of non-probability sampling and restriction to the safest accessible states in Sudan may limit the generalizability of findings and raise concerns about attributing observed access and satisfaction levels solely to the war. However, the widespread effects of the conflict—including displacement, economic collapse, healthcare workforce migration, and supply chain disruptions—even in safer areas, support cautious attribution. The absence of geographic identifiers limited our ability to analyze regional variations. Multimorbidity was included but not analyzed separately, potentially masking its cumulative impact. The conflict context may have influenced participant responses, introducing bias. The lack of qualitative data restricted deeper exploration of patient experiences, and the absence of pre-war baseline data limits our ability to fully isolate war-related effects. Future research should address these gaps for a more comprehensive understanding.

## Conclusion

This study describes substantial challenges in accessing and satisfaction with healthcare services among chronic disease patients during the Sudan armed conflict. More than one-third of participants reported visiting health facilities only once a year or not at all, indicating poor follow-up care. Over half of the patients reported that the ongoing conflict negatively affected their ability to access services, and one-fifth expressed dissatisfaction with the quality of healthcare provided.

To address the access challenges highlighted in this study, we recommend strengthening the availability of healthcare personnel and enhancing patient trust through capacity-building initiatives, improved supervision, and consistent service delivery. Policymakers should consider expanding outreach and mobile clinics to reduce physical and financial barriers, especially for internally displaced and low-income populations. Promoting community-based awareness about available services may also improve perceived ease of access and satisfaction. Given the ongoing conflict, adaptive health strategies that prioritize continuity of care, especially in relatively safer areas, are crucial. Additionally, future health interventions should be informed by real-time community feedback to ensure responsiveness and resilience of the healthcare system under crisis conditions.

## Supplementary Information

Below is the link to the electronic supplementary material.

Supplementary Material 1

## Data Availability

“The datasets used and/or analysed during the current study are available from the corresponding author on reasonable request.”

## Abbreviations

STROBE: Strengthening the reporting of observational studies in epidemiology
SPSS: Statistical package for the social sciences
IDPs: Internally displaced persons
UNHCR: United Nations high commissioner for refugees
