# Supplementary material for: Access and satisfaction with healthcare services among chronic disease patients during the Sudan armed conflict: a cross-sectional study
Source: Confl Health. 2025 Aug 11;19:61. doi: 10.1186/s13031-025-00703-y (PMC12337450; doi:10.1186/s13031-025-00703-y)
Supplement: Supplementary file 1 — Supplementary Material 1 [file 13031_2025_703_MOESM1_ESM.docx]

**Access and satisfaction with healthcare services among chronic disease patients during the Sudan armed conflict: A cross-sectional study**

**The Questionnaire**

**Section 1: Demographic Information**

1. **Age:**
   - __________ years
2. **Sex:**
   - Male
   - Female
3. **Marital Status:**
   - Single
   - Married
   - Divorced
   - Widowed
4. **What is your highest level of education completed?**
   - Illiterate
   - Informal education (Khalwa)
   - Primary education
   - Secondary education
   - Bachelor’s Degree
   - Higher studies
5. **What is your current employment status?**

- Unemployed
- Self-employed (including freelancing)
- Student
- Government employee
- Private sector employee

1. **What is your current living situation?**

- Living with family or friends
- Renting a house or apartment
- Owning a house or apartment
- Temporary housing/shelter
- Living in a refugee/Internally displaced camp

1. **Are you an internally displaced person (IDP)?**

- Yes
- No

1. **How would you classify your family's socioeconomic status?**

- Low
- Below average
- Average
- More than average
- High

1. **Have you ever been diagnosed with any of the following chronic diseases? (Select all that apply)**

- Diabetes Mellitus
- Systemic Hypertension
- Cardiovascular Diseases (Ischemic Heart Disease, Stroke)
- Respiratory Diseases (Chronic Obstructive Pulmonary Disease, Asthma, Chronic Respiratory Infections)
- Kidney Diseases (Chronic Kidney Disease)
- Cancers (Breast Cancer, Cervical Cancer, Colorectal Cancer, Liver Cancer, Thyroid Cancer)
- Musculoskeletal Disorders (Arthritis, Osteoporosis)
- Infectious Diseases (Tuberculosis, Leishmaniasis, Hepatitis B, Hepatitis C, Schistosomiasis, HIV/AIDS)

**Section 2: Access and satisfaction with healthcare services**

1. **How often do you visit a healthcare facility for regular check-ups during the current war?**
   - (1) Never
   - (2) Rarely (Once a year or less)
   - (3) Sometimes (Every 4–6 months)
   - (4) Frequently (Every 2–3 months)
   - (5) Very frequently (Monthly or more)
2. **How easy is it to get to the nearest health facility from your place of residence during the current war?**
   - (1) Very hard (More than 10 km away)
   - (2) Hard (5–10 km away)
   - (3) Moderate (1–5 km away)
   - (4) Easy (Less than 1 km away)
   - (5) Very easy (Within walking distance)
3. **How often do you have access to healthcare services when you need them during the current war?**
   - (1) Never
   - (2) Rarely
   - (3) Sometimes
   - (4) Often
   - (5) Always
4. **How do you assess the availability of healthcare personnel (doctors, nurses, etc.) at the facility you visit during the current war?**
   - (1) Very weak
   - (2) Weak
   - (3) Moderate
   - (4) Good
   - (5) Excellent
5. **How satisfied are you with the quality of care provided during your healthcare visits during the current war?**
   - (1) Very unsatisfied
   - (2) Unsatisfied
   - (3) Neutral
   - (4) Satisfied
   - (5) Very Satisfied
6. **How confident are you in your healthcare providers’ ability to manage your health during the current war?**
   - (1) Not Confident
   - (2) Slightly Confident
   - (3) Neutral
   - (4) Confident
   - (5) Very Confident
7. **To what extent has the conflict affected your ability to access healthcare services?**
   - (1) No effect
   - (2) Little effect
   - (3) Moderate effect
   - (4) Large effect
   - (5) Very large effect
